# Oral health knowledge, practice, and oral health status among rohingya refugees in Cox’s Bazar, Bangladesh: A cross-sectional study

**DOI:** 10.1371/journal.pone.0269359

**Published:** 2022-06-15

**Authors:** Sreshtha Chowdhury, Simanta Roy, Mehedi Hasan, Asif Al Sadique, Tariful Islam, Mehedi Hasan, Md. Yeasin Arafat, Md. Atiqur Rahman Bhuiyan, A. M. Khairul Islam, Omar Khalid, Ramisha Maliha, Mohammad Ali Hossain, Mohammad Lutfor Rahman, Mohammad Hayatun Nabi, Mohammad Delwer Hossain Hawlader

**Affiliations:** 1 Department of Public Health, North South University, Dhaka, Bangladesh; 2 Public Health Professional Development Society (PPDS), Dhaka, Bangladesh; 3 Ibn Sina Medical College Hospital, Kallyanpur, Dhaka, Bangladesh; 4 Institute of Statistical Research and Training (ISRT), University of Dhaka, Dhaka, Bangladesh; International Medical University, MALAYSIA

## Abstract

Oral health is a critical component of human health but is sometimes forgotten, particularly during humanitarian crises. This research aimed to ascertain the state of oral health among Rohingya refugees living in one of the largest refugee camps and evaluate their knowledge and practice of oral health. A multicenter cross-sectional survey was conducted among 477 participants from July to September 2021 using a structured questionnaire. There were 34 Rohingya camps and out of those 14 camps were accessible for data collection. The study participants were between 18–82 years residing in the refugee camps under Cox’s Bazar. The majority of participants (53.88%) were female and between the ages of 25 and 45. Around 46.12% of respondents did not have basic oral health knowledge, while 53.67% were in need of dental care. Nearly half of the participants demonstrated poor oral health practices. Participants’ age and educational level were positively associated with oral health knowledge (p = 0.02 and p<0.001). Furthermore, the knowledge level was positively associated with oral health practice (p = .025). Participants with a history of teeth pain and discomfort in the last 12 months were ten times more likely to seek treatment (OR = 9.93, CI: 5.591–17.64). The study indicated a growing demand for dental care among Rohingya refugees staying in Bangladesh. To reduce the severity of oral health issues, use of minimally invasive restorative procedures can be suggested in camps. New oral health promotion campaigns should be emphasized and proper education, ideally in their original language, can be beneficial.

## Introduction

Oral health is a highly significant component of human health but is often overlooked, especially in humanitarian emergencies like refugee crises. In almost every humanitarian catastrophe, emergency services take precedence over dental care. In 2013, a study in Australia on Refugees identified uncertainty regarding child oral hygiene habits and inadequate oral health literacy that affected children’s oral health status. This study explored the socio cultural determinants influencing child oral health among refugees [1]. Earlier studies performed by Ghiabi et al.^2^ found that uncorrected dental decay and gingivitis were prevalent among refugees in 85% and 98% of cases, respectively. Therefore, it is evident that oral health issues have been prevalent among any refugee population.

Refugees are defined by the United Nations High Commissioner for Refugees (UNHCR) as "someone who is unable or unwilling to return to their place of origin because of a well-founded fear of persecution on account of ethnicity, religion, nationality, membership in a specific social group, or political opinion [2]." To escape violence and oppression, Rohingya people fled the Rakhaine State of Myanmar. Almost a million took shelter in 34 refugee camps in the Cox’s Bazar district of Bangladesh [3]. The Rohingya refugee camps are overcrowded and have poor sanitary standards, putting their health in danger [4], and oral health is no exception. Oral health problems include several common risk factors that contribute to chronic illnesses like cancers [5], cardiovascular diseases [6]. The frequency of dental disease has been significant among refugees [7]. Prolonged discomfort from defective teeth might indicate severe oral health issues. Oral discomfort can also encourage lower consumption of food, which can cause malnutrition and ultimately lead to more significant health problems [8,9]. A study among Rohingya refugees in India found that 83.92% of people aged 15 and above had dental caries. Nearly all study participants had gingival bleeding and periodontal disease. The prevalence of dental caries was 85.23% of children under 15. Periodontal disease and gingival bleeding also had a high prevalence among the Rohingya refugees in India [10]. The lack of access to restorative and preventative dental care is one of the primary drivers of these people’s poor oral health. Poor dental hygiene, nutritional variables, sociocultural issues, and lack of health literacy are other contributors. Cultural variables might also significantly determine access to the oral hygiene facilities and oral hygiene behaviors among these people [11].

Oral health knowledge is considered fundamental for the development of healthy habits, and studies have shown that increasing knowledge is associated with improved oral health [12]. Optimal health-related practices are more likely to be adopted if a person feels more in control of their health as a result of a greater awareness of diseases and their causation [13,14]. Traditional behavior change theory states that if we are successful in transmitting knowledge into the communal environment, it will usually result in an improvement in the attitudes and health-related behaviors of the wider population [13].

Due to the high population density and inadequate resources and services in the camps, accessing healthcare facilities becomes difficult at times. As a result, the population is deprived of critical oral and physical health services, exposing them to a range of health and oral diseases [10]. The COVID-19 pandemic has also added stress, impairing their emotional, physical, and dental health. Eighty health partners provide vital health care in this humanitarian setting, but few works on the refugees’ oral health [15]. From our knowledge there were limited studies done among the Rohingya migrants and refugees to explore their existing oral health status as well as their knowledge and practice towards it. Therefore, our study was carried out to assess the knowledge, practice, and oral health status among the Rohingya refugees in Cox’s Bazar, Bangladesh.

## Method

### Study setting

Our study was conducted from July to September 2021 in Teknaf and Ukhiya, Cox’s Bazar, Bangladesh. For data collection, 14 refugee camps were accessible out of 34 camps. According to the UNHCR’s population factsheet for 31 December 2020, the camps housed approximately 180,616 adults (18 years and older). In accordance with our study’s inclusion criteria, the number of participants from each camp was determined by the following formula: n_e_ = n x N_e_/N; (where n_e_ = Number of sample from each camp, n = Total sample, N = Total population, N_e_ = Population of each camp.)

### Study design

In this multicenter cross-sectional study, the individuals aged 18 years or older who were living in the Cox’s Bazar Rohingya camp were included. Before the study, a questionnaire was developed by vigorously reviewing the similar published literature. After developing the questionnaire, it was translated in participant’s native language with the help of two independent translators [16]. The content validity of the questionnaire was confirmed by independent assessments by a panel of three Associate Professor level dental surgeons from three reputable medical institutions in Bangladesh. The experts were properly informed about the aims and objectives of the study but the identification of the authors were kept confidential. The questionnaire was then piloted with 19 individuals to verify its appropriateness. Additionally, we determined the questionnaire’s reliability using Cronbach’s alpha and found an overall score of 0.84, indicating a high internal consistency. Cronbach’s alpha values for the "Knowledge" and "Practice and Self-reported oral health status" subscales were 0.90 and 0.69, respectively. Researchers and trained volunteers performed the interview using a printed structured questionnaire and the KOBO Collect program [17].

### Measures

A five-point Likert scale was used to score the participants’ knowledge of oral health. We assigned a score of 5 to "Strongly Agree," 4 to "Agree," 3 to "Neither agree nor disagree," 2 to "Disagree," and 1 to "Strongly disagree".

For assessment of oral health practice, four questions were asked with different options. For assessment of daily brushing frequency, the options and scores were "Once a day" = 1, "Twice a day" = 2," Do not brush daily" = 0. Similarly, In case of tooth-cleaning material "Toothbrush" = 2 and "Charcoal/Toothpick/Others" = 0. "Miswak" and "Dental floss/Thread" are helpful but cannot replace tooth brush hence these two were scored 1 [18,19]. In case of using toothpaste "Yes" = 1, "No" = 0. For dental problems from where they seek treatment "Dentist" = 2, "General Practitioner" = 1, "Medical assistant/Dental assistant/Ethno medicine" = 0. The maximum achievable score was 7. After taking the expert opinion, we categorized the participants with the scores above three as "healthy practice" and those with three or below as "unhealthy practice."

There were 12 statements with five options for assessing the oral health status. For statements such as "Difficulty with biting food," options were "2–3 times every month" = score 3, "Once in a month" = score 2, "2–3 times in 12 months" = score 1, "Never" = score 0, "Don’t know” = score 0. The overall total possible score was 36.

### Ethics statements

The study was conducted according to the guidelines of the Declaration of Helsinki and approved by the Institutional Review Board (Ethics committee) of North South University (Ref: 2021/OR-NSU/IRB/0703). Before interviewing each subject, the participants were clearly briefed about the aims, procedures of the research. Verbal consent was taken considering the low literacy level of the participants [20]. Maintaining physical distance for COVID-19 was another consideration for verbal consent. This use of verbal consent was also approved by the Institutional Review Board North South University. Sufficient time was allowed for questions to be asked and answered, both by the participants and by the volunteers to ensure the participant comprehended the consent information. Once the participant consented to voluntary participation, a note was written in the study participant’s record. The interviewers made a concerted effort to maintain the anonymity of the respondents by conducting private interviews in their homes or during their visits to health care facilities, barring outsiders.

### Statistical analysis

The data were analyzed using STATA software version 16. Frequencies and percentages were used to classify and explain all variables. The chi-square test was performed to investigate the bivariate relationship between categorical variables, and logistic regression models were fitted to identify factors associated with outcomes.

## Result

The majority (45.78%) of the participants were between 25 and 45, with females representing 53.88%. The study participants were all Muslims, with 82.60% married. About 72% were old migrants (migrated more than 42 months back), and 64.57% were unemployed before migration. Among the migrant, 85.74% became employed after relocating to Bangladesh. 45.70% were uneducated, whereas 33.33% had completed 1 to 5 years of education and 20.96% had studied more than five years **(Table 1)**.

**Table 1:** Demographic characteristics of the participants and its association with oral health knowledge (N = 477).

Variable			Knowledge		
Demographic information	N	%	Poor N (%)	Good N (%)	P value
			220(46.12)	220(46.12)	
**Age**					
<25 years	138	29.11	75(54.35)	63(45.65)	**0.020** **
25–45 years	217	45.78	99(45.62)	118(54.38)	
>45 years	119	25.11	44(36.97)	75(63.03)	
**Gender**					
Male	220	46.12	94(42.73)	126(57.27)	0.170
Female	257	53.88	126(49.03)	131(50.97)	
**Marital status**					
Never married	83	17.40	41(49.40)	42(50.60)	0.510
Married	394	82.60	179(45.43)	215(54.57)	
**Past occupation**					
Unemployed	308	64.57	150(48.70)	158(51.30)	0.130
Employed	169	35.43	70(41.42)	99(58.58)	
**Present occupation**					
Unemployed	409	85.74	187(45.72)	222(54.28)	0.670
Employed	68	14.26	33(48.53)	35(51.47)	
**Years of education**					
Uneducated	218	45.70	92(42.20)	126(57.80)	**<0.001** ***
1 to 5 years	159	33.33	90(56.60)	69(43.40)	
>5 years	100	20.96	38(38.00)	62(62.00)	
**Migration status**					
Recent migrant (≤42 months)	130	27.25	54(41.54)	76(58.46)	0.220
Old migrant (>42 months)	347	72.75	166(47.84)	181(52.16)	
**Oral health-related personal information**					
**Number of natural teeth**					
<20	55	11.53	28(50.91)	27(49.09)	0.450
20 or more	422	88.47	192(45.50)	230(54.50)	
**Teeth pain or discomfort in last 12 months**					
Yes	215	45.07	87(40.47)	128(59.53)	**0.030** **
No	262	54.93	133(50.76)	129(49.24)	
**Artificial teeth**					
Yes	27	5.66	2(7.41)	25(92.59)	**<0.001** ***
No	450	94.33	218(48.44)	232(51.56)	

** = Significant

*** = Highly significant.

Regarding oral health-related personal information, 88.47% had 20 or more natural teeth, and about 45% experienced dental pain or discomfort in the previous 12 months. Only 5.66% of the individuals had artificial teeth. Regarding knowledge of the participant, for the ten questions, the mean score was 42.32 **(Table 2)** whereas the maximum achievable score was 50. Those scored above the mean (≥42) were categorized as having "Good knowledge." A score below 42 was classified as having "Poor knowledge". The prevalence of participants with good and poor knowledge was 53.88% and 46.12%, respectively.

**Table 2:** Oral health knowledge of the participants (N = 477).

Question	Strongly disagree	Disagree	Neither agree nor disagree	Agree	Strongly agree	Mean score
	Score 1	Score 2	Score 3	Score 4	Score 5	(Overall 42.32)
1. Teeth are an important part of your Body	1(0.21)	0(0.00)	20(4.19)	202(42.35)	254(53.25)	4.48
2. Keeping your mouth clean and healthy is good for health	1(0.21)	3(0.63)	30(6.30)	20(43.70)	234(49.16)	4.40
3. Dental disease may cause other health problem	2(0.42)	11(2.31)	95(19.92)	166(34.80)	203(42.56)	4.16
4. Two-time brushing daily is good for oral health	2(0.42)	8(1.68)	86(18.03)	195(40.88)	186(38.99)	4.16
5. Gum bleeding is a bad sign for oral health	3(0.63)	7(1.47)	61(12.79)	232(48.64)	174(36.48)	4.18
6. Bad breath is a bad sign for oral health	3(0.63)	12(2.52)	99(20.75)	192(40.25)	171(35.85)	4.08
7. Smoking is harmful for oral health	2(0.42)	4(0.84)	62(13.00)	229(48.01)	180(37.74)	4.22
8. Chewing tobacco is harmful for oral health	5(1.05)	4(0.84)	85(17.82)	196(41.09)	187(39.20)	4.17
9. Using toothbrush is good for oral health	3(0.63)	4(0.84)	63(13.21)	214(44.86)	193(40.46)	4.24
10. Using toothpaste is good for oral health	3(0.63)	4(0.84)	72(15.09)	210(44.03)	188(39.41)	4.21

In terms of oral health practice, their final score was categorized as: 0 = Normal, 1–12 = Mild, 13–24 = Moderate, and 25–36 = Severe. Furthermore, the "Normal" group was categorized as "Healthy" and "Mild," "Moderate" and "Severe" groups were categorized as" Needs dental care" to signify the oral health status of the study population. The prevalence of "healthy practice" was 52.20%, and "unhealthy practice" was 47.80% **(Table 3)**. Among the participants, the prevalence of mild, moderate, and severe oral health problems were 29.56%, 16.35%, and 7.76%, respectively.

**Table 3:** Prevalence of oral health knowledge, practice, and self-reported oral health status (n = 477).

	N	%
**Knowledge**		
Poor knowledge	220	46.12
Good knowledge	257	53.88
**Practice**		
Unhealthy practice	249	52.20
Healthy practice	228	47.80
**Self-reported Oral Health Status**		
Healthy	221	46.33
Needs dental care	256	53.67
Mild problem	141	29.56
Moderate problem	78	16.35
Severe problem	37	7.76

We performed bivariate analysis and presented the unadjusted result in T**ables 1**and **4,** which identified several potential factors associated with the knowledge, practice, and oral health status of the Rohingya population. **Table 1**showed that age, years of education, teeth pain or discomfort in the last 12 months, and presence of artificial teeth were significantly associated with oral health knowledge. Simultaneously, age (p<0.001, p<0.001) marital status (p<0.001, p<0.001), years of education (p<0.001, p<0.001), teeth pain or discomfort in the last 12 months (p<0.001, p<0.001) were significantly associated with both self-reported oral health status and oral health practice. On the other hand, gender (p<0.001), past occupation (p<0.001), and migration status (p<0.001) were only associated with oral health status. In contrast, the number of natural teeth (p<0.001) and the presence of artificial teeth (p = .001) were only related to oral health practice.

**Table 4:** Self-reported oral health status and practice and their associated factors (N = 477).

Variable	Self-reported oral health status			Oral health practice		
**Demographic information**	Healthy N (%)	Needs dental care N (%)	P value	Unhealthy Practice N (%)	Healthy Practice N (%)	P value
	221(46.33)	256(53.67)		249(52.20)	228(47.80)	
**Age**						
<25 years	105(76.09)	33(23.91)	**<0.001** ***	48(34.78)	90(65.22)	**<0.001** ***
25–45 years	96(44.24)	121(55.76)		110(50.69)	107(49.31)	
>45 years	19(15.97)	100(84.03)		89(74.79)	30(25.21)	
**Gender**						
Male	76(34.55)	144(65.45)	**<0.001** ***	118(53.64)	102(46.36)	0.562
Female	145(56.42)	112(43.58)		131(50.97)	126(49.03)	
**Marital status**						
Never married	55(66.27)	28(33.73)	**<0.001** ***	25(30.12)	58(69.88)	**<0.001** ***
Married/Divorced	166(42.13)	228(57.87)		224(56.85)	170(43.15)	
**Past occupation**						
Unemployed	171(55.52)	137(44.48)	**<0.001** ***	159(51.62)	149(48.38)	0.733
Employed	50(29.59)	119(70.41)		90(53.25)	79(46.75)	
**Present occupation**						
Unemployed	193(47.19)	216(52.81)	0.357	220(53.79)	189(46.21)	0.089
Employed	28(41.18)	40(58.82)		29(42.65)	39(57.35)	
**Years of education**						
Uneducated	73(33.49)	145(66.51)	**<0.001** ***	156(71.56)	62(28.44)	**<0.001** ***
1 to 5 years	88(55.35)	71(44.65)		62(38.99)	97(61.01)	
> 5 years	60(60.00)	40(40.00)		31(31.00)	69(69.00)	
**Migration status**						
Recent migrant (≤42 months)	98(75.38)	32(24.62)	**<0.001** ***	75(57.69)	55(42.31)	0.142
Old migrant (>42 months)	123(35.45)	224(64.55)		174(50.14)	173(49.86)	
**Oral health-related personal information**						
**Number of natural teeth**						
<20	27(49.09)	28(50.91)	0.663	45(81.82)	10(18.8)	**<0.001** ***
20 or more	194(45.97)	228(54.03)		204(48.34)	218(51.66)	
**Teeth pain or discomfort in last 12 months**						
Yes	32(14.88)	183(85.12)	**<0.001** ***	141(65.58)	74(34.42)	**<0.001** ***
No	189(72.14)	73(27.86)		108(41.22)	154(58.78)	
**Artificial teeth**						
Yes	11(40.74)	16(59.26)	0.549	6(22.22)	21(77.78)	**0.001** **
No	210(46.67)	240(53.33)		243(54.00)	207(46.00)	
**Oral health knowledge**						
Poor knowledge	103(46.82)	117(53.18)	0.844	127(57.73)	93(42.27)	**0.025** **
Good knowledge	118(45.91)	139(54.09)		122(47.47)	135(52.53)	

P = P value

*** = highly significant

** = significant.

### Oral health knowledge

In a multivariate logistic regression model, we included the potential variables from the bivariate analysis. Participants aged greater than 45 years have more knowledge (OR = 2.34) about oral health than those aged less than 25 years (OR = 2.34, 95% CI: 1.207–4.531). Participants who studied for 1–5 years had 57% less knowledge regarding oral health than those who studied for more than five years(OR = .43, 95% CI:.249-.753). Individuals with artificial teeth had nearly 9 times higher knowledge about oral health than those without artificial teeth (OR = 9.15, 95% CI: 2.08–40.32). Old migrants had 52% less knowledge than the recent migrants (OR = .48, 95% CI: .297-.800), and nearly 3.5 times better knowledge was observed among the participants with severe oral problems (OR = 3.67, 95% CI: 1.394–9.656). Participants with healthy oral health practice were 2.16 times more knowledgeable than those with unhealthy practice (OR = 2.16, 95% CI: 1.373–3.385) **(Table 5).**

**Table 5:** Logistic regression of oral health knowledge, practice, and self-reported oral health status (N = 477).

	Oral health knowledge				Oral health practice				Self-reported oral health status			
Variable	AOR	p-value	95% CI		AOR	p- value	95% CI		AOR	p- value	95% CI	
**Age**												
< 25 years	1				1				1			
25–45 years	1.496	0.114	.907	2.466	.896	0.687	.525	1.529	3.508	**<0.001** ***	1.913	6.433
> 45 years	2.339	**0.012** **	1.207	4.531	.469	**0.042** **	.226	.973	8.101	**<0.001** ***	3.545	18.51
**Years of schooling**												
>5 years	1				1				1			
1–5 years	.433	**0.003** **	.249	.753	.906	0.749	.497	1.654	1.712	0.125	.861	3.401
Uneducated	.603	0.094	.334	1.09	.30	**<0.001** ***	.161	.56	.951	0.892	.463	1.953
**Teeth pain and discomfort in last 12 months**												
No	1				1				1			
Yes	1.255	0.410	.731	2.156	.939	0.830	.526	1.675	9.931	**<0.001** ***	5.591	17.64
**Artificial teeth**												
No	1				1				1			
Yes	9.157	**0.003** **	2.08	40.32	4.548	**0.007** **	1.518	13.622	.38	0.064	.137	1.056
**Migration status**												
Recent migrant (≤42 months)	1				1				1			
Old migrant (>42 months)	.487	**0.004** **	.297	.8	2.739	**<0.001** ***	1.604	4.676	9.488	**<0.001** ***	4.954	18.171
**Severity**												
Normal	1				1							
Mild	.71	0.230	.405	1.243	.483	**0.016** **	.267	.873				
Moderate	1.265	0.545	.591	2.71	.304	**0.003** **	.137	.673				
Severe	3.669	**0.008** **	1.394	9.656	.015	**<0.001** ***	.002	.125				
**Practice**												
Unhealthy	1								1			
Healthy	2.156	**0.001** **	1.373	3.385					.353	**<0.001** ***	.203	.614

AOR = Adjusted odds ratio, CI = Confidence interval

** = Significant

*** = Highly significant.

### Oral health practice

**Fig 1:**
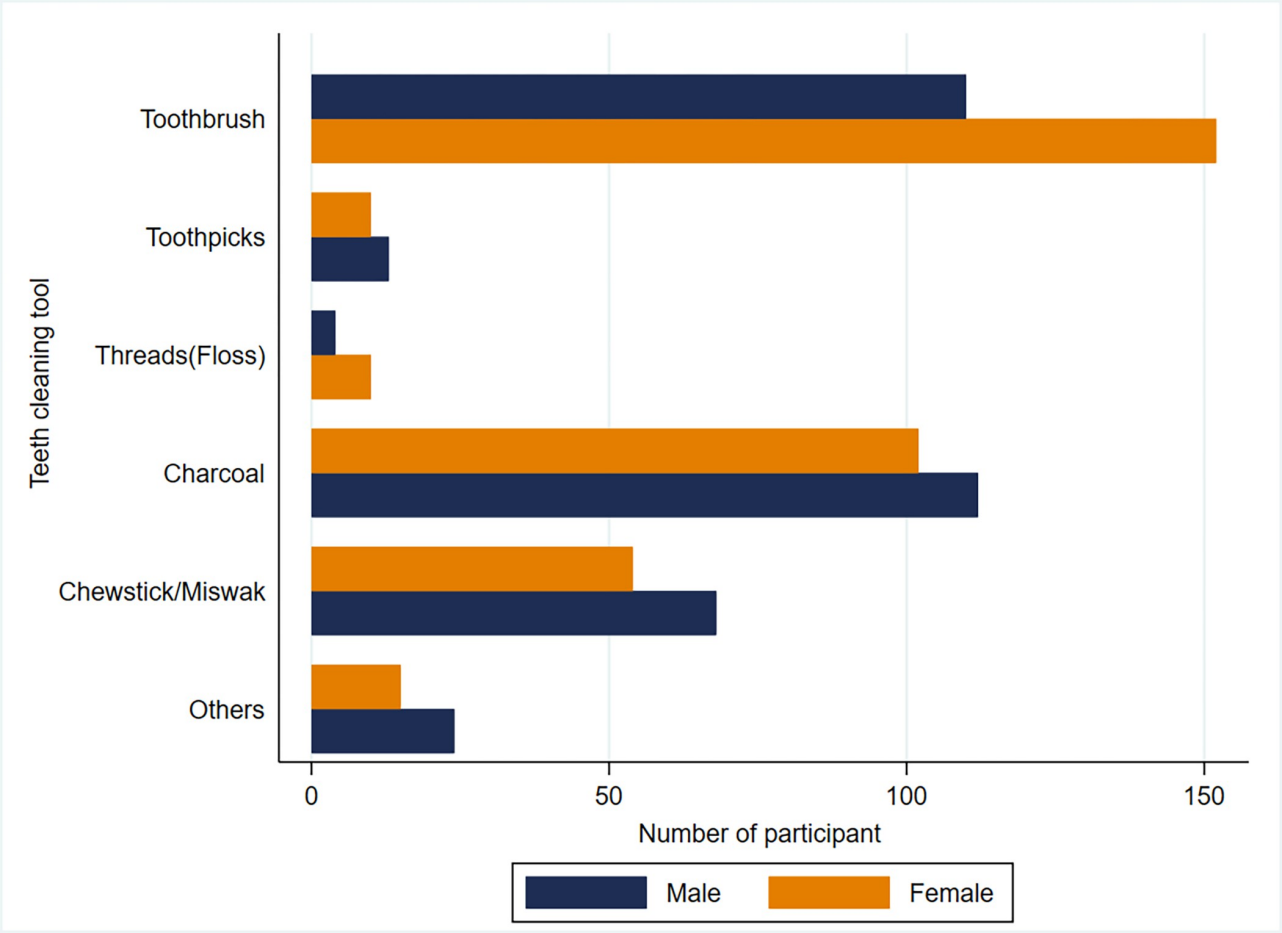
Bar chart showing teeth cleaning tool by gender of the participants.

**Fig 2:**
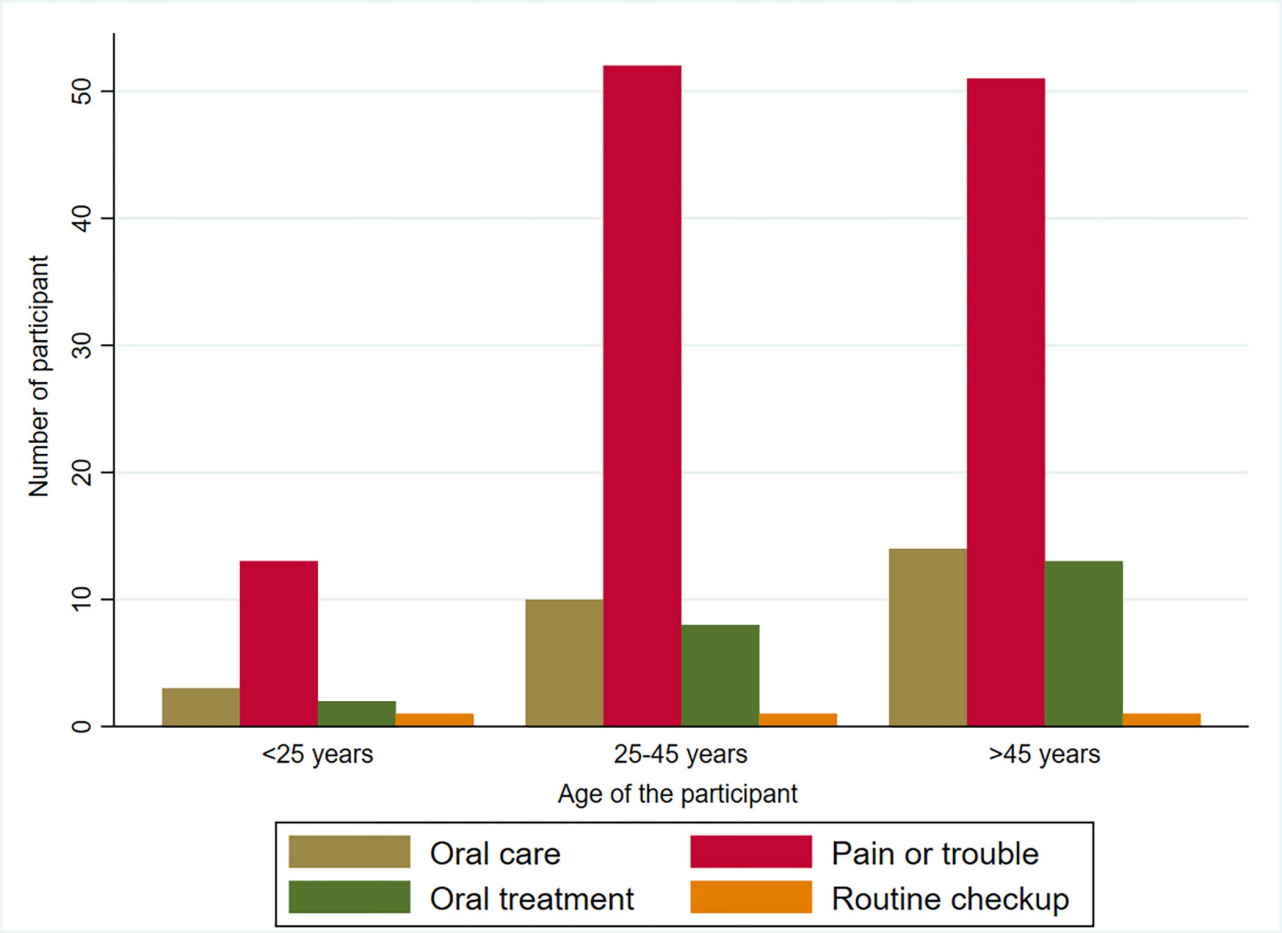
Bar showing the reason for last dental treatment/appointment.

Participants aged more than 45 years had 54% less tendency to healthy practice regarding oral health than those aged below 25 years (OR = .46, 95% CI: O.226-0.973). Uneducated participants had a 70% less tendency of healthy practice regarding oral health than those studied more than five years (OR = 0.30, 95% CI: .161–0.56). Old migrants had nearly 2.5 times better practice than those who migrated recently (within last 42 months) (OR = 2.73, 95% CI: 1.604–4.676). Participants with artificial teeth had 4.5 times more intention of healthy practice than those without artificial teeth (OR = 4.56, 95% CI: 1.518–13.622). Participants with healthy oral health practice had 65% less need of treatment (OR = .353, 95% CI: .203-.614). Participants with good oral health knowledge had two times better practice than those with poor knowledge (OR = 2.156, 95% CI: 1.373–3.385) **(Table 5).** Tooth cleaning tools are inseparable parts of oral health practice. Fig 1 shows the high use of toothbrushes, especially among females, followed by charcoal and miswak. Moreover, most of the respondents considered dental treatment only for pain or trouble rather than regular check-up (Fig 2).

### Self-reported oral health status

Regarding self-reported oral health status, participants between the ages 25–45 years, and those beyond 45 were 3.50 and 8.10 times in need of dental treatment than those under 25 (OR = 3.50 and OR = 8.10, 95% CI:1.913–6.433 and CI: 3.545–18.51 respectively). Participants who reported dental pain in the previous 12 months were 9.93 times more likely to need treatment than those without such complaints (OR = 9.93, 95% CI:5.591–17.64). Old migrants (who arrived in Bangladesh more than 42 months ago) had a 9.48-fold more demand for dental care than the new migrants (OR = 9.48, 95% CI: 4.95–1817).

## Discussion

The status of one’s oral health has a significant impact on one’s overall health and quality of life. On the other hand, poor oral health affects physical, psychological, and social well-being. It is common for the migrant population to live a life of poverty and despair due to a scarcity of resources such as housing, education, healthcare, and work prospects which eventually predisposes this disadvantaged society to more significant health risks and poor dental health regularly [21,22]. Even though the Rohingya camp in Bangladesh is currently the world’s largest refugee camp, there has been little attention and research on the oral health issues of the Rohingya population [23].

According to our study’s findings, roughly half of the respondents had good oral health knowledge, which is similar to the results of a study conducted by Solyman et al. on refugees in Germany [24]. Additionally, the prevalence of healthy practice was seen only in less than half (47.80%) of the respondents, and this might result from a lack of dental clinics or access to them on native land or after relocation to the area where they are now residing. Comparable results were seen in another oral health-related study on heart disease patients [25].

In our study, the aged participants were found to have significantly more oral health knowledge than the younger folks. Similar associations were described by Pinnamaneniet al. [26]. However, contrasting findings have been discussed by Ramesh et al.in an oral health-related study on parents of infants [27]. Studies conducted by Basavaraj et al. [21] and Smyth et al. [28], our study found that a higher level of education is related to higher oral health knowledge and healthy practices. This might be due to greater competence among educated people in detecting a problem, seeking assistance, locating appropriate resources, comprehending the issue, and making a choice.

Refugees who arrived in Bangladesh from Myanmar more than 3.5 years back had healthy practices and better oral health status than the more recent migrants despite their poor knowledge. This might be a possible effect of migrants’ longer exposure to teeth cleaning equipment, better health facilities and awareness programs This finding is further reinforced by studies on refugees in Germany and Vietnamese migrants [24,29]. Contradicting data has been provided in a Burundian refugee camp survey, where the authors hypothesized that individuals who had been in the camp for a longer span of time had developed complacency in comparison to recent arrivals, resulting in unhealthy practices.

Another strong correlation was seen between having artificial teeth and their oral health knowledge and practice. Those with artificial teeth demonstrated 9-fold better knowledge and nearly five-fold healthier practice, probably due to their previous encounter with the dental practitioner. In an Indian study, individuals with dental implants showed appropriate knowledge, but they lacked sufficient oral hygiene practices [30]. It was speculated that such disinterest in implant or denture users resulted from neglect.

Knowledge is necessary to form prevention beliefs, cultivate good attitudes, and promote positive thoughts toward illness. This was mirrored in the practice of the majority of our survey respondents. This research showed that participants with adequate knowledge of oral health practiced twice healthier than those with insufficient knowledge. A study conducted by Wahengbam et al. also had similar findings [31]. Besides, a healthy oral health practitioner had a 65% reduced likelihood of requiring dental care as the healthy practice has a protective influence on self-reported oral health [32]. Similar findings were previously suggested by Liu et al. [33].

People with dental pain need dental treatment more than healthy people [33]. Individuals with dental pain in the previous 12 months were almost 10 times more likely to require dental treatment. Asking people about dental pain might be considered as a possible screening approach to identify individuals in refugee camps who need dental treatment in order to assure appropriate support in a resource-limited setting.

To our knowledge, this was the first ever research to investigate refugee oral health status in Bangladesh. Besides, it was a multicenter study, which included participants from the major areas of the refugee camp. Moreover, the face-to-face interview and use of their native language enabled us to draw a clear picture of their oral health. However, the study has certain limitations. The data were self-reported, where there is a chance of recall bias. We could not perform dental checkups on the participants, which would have helped us better understand their oral health status. All face-to-face interviews were performed during the daytime while males were outside the home, so most participants were females.

## Conclusion

According to the findings of the study, it is essential for the development organizations who work for the Rohingya community to undertake long-term measures to increase access to oral health care. It is also necessary to adopt minimally invasive restorative treatments in camps in order to minimize the severity of oral health problems. In order to effectively promote oral health programs among Rohingyas, it is necessary to provide precise instructions on beneficial oral health habits in their local language. Additional educational efforts should be undertaken, and those in positions of responsibility should make measures to ensure that these underserved communities get adequate dental treatments.

## Supporting information

S1 File (DTA)
